# Location, Location, Location: The Impact of Migratory Heterogeneity on T Cell Function

**DOI:** 10.3389/fimmu.2013.00311

**Published:** 2013-10-08

**Authors:** Bas J. G. Baaten, Andrea M. Cooper, Susan L. Swain, Linda M. Bradley

**Affiliations:** ^1^Infectious and Inflammatory Diseases Center, Sanford-Burnham Medical Research Institute, La Jolla, CA, USA; ^2^Trudeau Institute, Saranac Lake, NY, USA; ^3^Department of Pathology, University of Massachusetts Medical School, Worcester, MA, USA

**Keywords:** T cell, heterogeneity, subset, migration, memory, cellular, immunity, motility

## Abstract

T cell migration is crucial for an effective adaptive immune response to invading pathogens. Naive and memory T cells encounter pathogen antigens, become activated, and differentiate into effector cells in secondary lymphoid tissues, and then migrate to the site(s) of infection where they exert effector activities that control and eliminate pathogens. To achieve activation, efficient effector function, and good memory formation, T cells must traffic between lymphoid and non-lymphoid tissues within the body. This complex process is facilitated by chemokine receptors, selectins, CD44, and integrins that mediate the interactions of T cells with the environment. The expression patterns of these migration receptors (MR) dictate the tissues into which the effector T cells migrate and enable them to occupy specific niches within the tissue. While MR have been considered primarily to facilitate cell movement, we highlight how the heterogeneity of signaling through these receptors influences the function and fate of T cells *in situ*. We explore what drives MR expression heterogeneity, how this affects migration, and how this impacts T cell effector function and memory formation.

## Introduction

In this review, we focus on T cell heterogeneity defined as the variation in the expression of migration receptors (MR), including chemokine receptors (CCRs), selectins, CD44, and integrins. The heterogeneity of effector T cells is evident during clonal expansion, differentiation, functional development, and transition to memory and is influenced by interactions with dendritic cells (DC), the tissue environment, and the inflammatory status. MR expression heterogeneity not only governs T cell migration to specific niches in the lymphoid tissues or in non-lymphoid sites of infection and inflammation, but also allows for contextual communication through engagement of the microenvironment at these sites to facilitate T cell differentiation and effector function.

## Setting the Scene: Motility Enables the Initiation of the T Cell Response

For pathogens that enter and infect sites that interface with the external environment, such as the lung, skin, and intestinal tract, a complex interplay of innate and adaptive immune responses is required to achieve pathogen control and/or clearance. A functional T cell response is a crucial component of effective immunity to infection with pathogens and is influenced by a multitude of factors that include the microbe-specific mechanisms of host engagement, the site(s) of entry, and the virulence of the pathogen. Migration of T cells is crucial for an effective effector response to occur. For many intracellular pathogens, control and/or clearance depends upon the effector activities of CD8+ and CD4+ T cells that are programed to mediate type I responses characterized by cytolytic activity and production of cytokines, such as interferon (IFN)-γ and tumor necrosis factor (TNF)-α. These responses are initiated primarily by antigen-presenting DC that have migrated from the site of infection to the secondary lymphoid organs (SLO). In lymph nodes (LN), DC compartmentalize into the T cell zone along fibroblastic reticular cells that express the CCR7 ligands, CCL19 and CCL21 (1, 2). In systemic infections, responses are also initiated in the spleen, where DC enter from the blood into the marginal zone of the red pulp, and from there migrate into the T cell zone of the white pulp in response to CCR7 ligands. DC become functional as antigen-presenting cells (APC) by upregulating MHC molecules and co-stimulatory molecules in response to innate signals that include pathogen-induced toll-like receptors and type I IFN, as well as by signaling via MR in response to migration (3). Many pathogen-specific aspects dictate and influence the innate response (4), which in turn impacts the extent of effector development in the adaptive response through effects on macrophages, DC, and other innate immune cells.

Highly motile naïve T cells that themselves are continuously recirculating interact with DC after entry into SLO. T cell migration from the blood into different tissues is regulated by a general cascade of events that is initiated by engagement of endothelial cells. This interaction consists of rolling and tethering, followed by firm adhesion, spreading/crawling, and finally extravasation (5). For naïve T cells, it is well-established that L-selectin (CD62L), CCR7, and LFA (CD18, β_2_) are the key molecules that regulate entry into LNs, which occurs through high endothelial venules that present their respective ligands, PNAd, CCL21, and ICAM-1. Within the LN, naïve T cells undergo cytoskeletal rearrangements that support motility, which in combination with CCR signaling facilitates directional motility toward DC (6). Naïve antigen-specific T cells engage antigen-bearing DC in a progression of serial encounters that result in the upregulation of activation markers such as CD69, CD25, and CD44. To ensure maximal activation and early retention in the LN, expression of the sphingosine-1-phosphate receptor 1 (S1P1) is initially reduced thereby lowering the ability of the T cell to be responsive to blood S1P levels [reviewed in Ref. (7)]. When T cells engage DC, their motility is greatly reduced and extended contacts between T cells and DC facilitate optimal activation and differentiation. During this process, MR expression profiles change dramatically. The impact of MR heterogeneity on T cell migrational capacity and effector function will be discussed in more detail below.

## Induction of Migratory Heterogeneity during Effector T Cell Development

**Figure 1:**
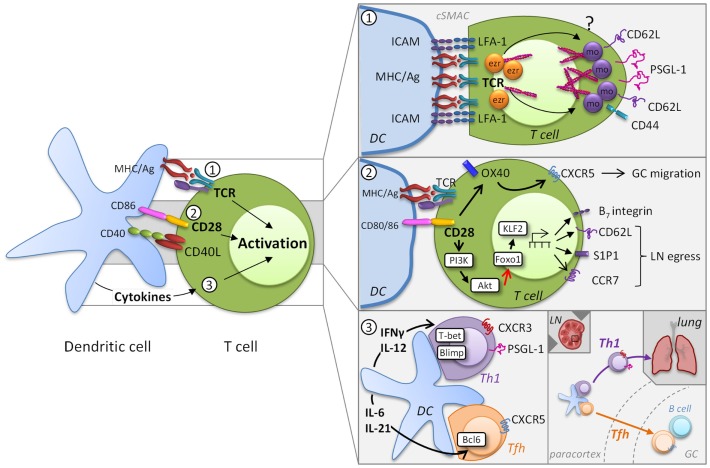
**Induction of migratory heterogeneity during priming**. MR phenotype is impacted by TCR engagement, the level of co-stimulation, and the cytokine milieu (*left*). MR play a direct role in the formation of the immunological synapse, but TCR signaling subsequently impacts MR expression [panel 1, adapted from Ref. (8)]. Cytoskeletal rearrangements that involve the actin-binding ezrin (ezr), radixin, and moesin (mo) proteins are necessary for TCR signaling complex polarization. The integrin LFA-1 forms a ring surrounding the cSMAC that supports prolonged T cell-DC engagement, while other MR become excluded from the cSMAC. This process is possibly due to differential polarization of ezrin and moesin. Co-stimulatory signaling through molecules also contributes to the migratory heterogeneity of T cells (panel 2). For example, CD28 controls migration through upregulation of OX40, which is instrumental for CXCR5 expression and T cell localization to germinal centers (GC). In addition CD28/TCR signaling activates the PI3K/AKT pathway, which inhibits Foxo1 leading to decreased KLF2 expression. Differential co-stimulation can impact the levels of CD62L, CCR7, and S1P1 and thereby regulate the egress of T cells into the circulation. Cytokines released by DC promote specific transcriptional profiles that introduce further MR heterogeneity (panel 3). DC-derived IL-12 induces expression of the transcription factor T-bet and determines a CD4+ Th1 or CD8+ effector transcriptional program that results in part in the expression of CXCR3 and PSGL-1, which contribute to homing to peripheral sites. Alternatively, induction of the Tfh-associated transcription factor Bcl6 by IL-6 and IL-21 results in the downregulation of PSGL-1 and increased expression of CXCR5, which allows these cells to migrate from the T cell zones in the paracortex into GC.

MR expression and ligation have important roles in T cell activation processes and thereby control effector T cell development in lymphoid tissues and non-lymphoid sites of infection and inflammation. At early stages in the development of effectors, MR heterogeneity is introduced by the differences in activation states of responding T cells that are largely determined by the degree of access to highly stimulatory APC. Here, we will explore the contribution of T cell receptor (TCR) affinity, the level of co-stimulation, and the cytokine milieu to MR expression and how they can influence MR heterogeneity during priming (Figure 1).

## TCR Signaling Induces MR Redistribution and *De Novo* MR Expression

Effective T cell activation depends on a dynamic interplay between TCR and peptide-MHC binding kinetics and the epitope density on the DC. MR play a direct role in the formation of the immunological synapse when engaging APC through actin rearrangement [reviewed in Ref. (8)]. Cytoskeletal rearrangements that involve the actin-binding ezrin, radixin, and moesin (ERM) proteins are necessary for T cell activation and IL-2 production (9, 10). To achieve TCR signaling complex polarization, MR that include CD44, CD62L, P-selectin glycoprotein (PSGL)-1, and ICAMs 1–3, become excluded from the central immunological synapse where the TCR and associated signaling molecules coalesce to form the central supramolecular activation cluster (cSMAC). The aforementioned MR become cross-linked to the actin cytoskeleton at the back of the cell, whereas the integrin LFA-1 forms a ring surrounding the cSMAC that supports prolonged T cell-DC engagement (11). Although little is known regarding the mechanisms by which T cells disengage from APC, once this occurs, T cells can interact with other cells via MR. For example, a recent study demonstrates that reciprocal ICAM-LFA interactions facilitate antigen-independent T cell–T cell synapses, which are required for the optimal generation of CD8+ effector T cell responses (12). These findings underscore that proper distribution and coordinated interplay of molecules in the TCR complex and MR are critical for full T cell activation.

The strength of TCR signaling represents a key checkpoint in the development of heterogeneous effector T cells. Strong stimulatory conditions lead to modulation of MR including upregulation of various integrins, CD44, and PSGL-1, with downregulation of CD62L and CCR7, a phenotype associated with the most highly functional effectors. This can, to some extent, be achieved by activating T cells with high affinity TCRs that can engage greater or distinct downstream signaling compared to low affinity TCRs (13, 14), and can result in proliferation versus cytokine production (13). However, for both CD4+ and CD8+ T cells, even individual naïve cell clones can give rise to a whole spectrum of heterogeneous effector phenotypes that can be influenced by antigen-dose and the duration of peptide-MHC binding for CD4+ T cells (15–17).

## Co-Stimulation during Priming Impacts MR Heterogeneity

Another major contributor to T cell activation and modulation of MR expression is the availability of co-stimulatory signaling through molecules such as CD28 that are not only essential for T cell proliferation, differentiation, and survival, but also impact T cell migration (Figure 1, panel 2). The amount of co-stimulation received and the individual co-stimulatory receptor(s) involved in T cell activation can also contribute to the migratory heterogeneity of T cells responding to a pathogen. For example, while CD28 and CTLA4 engagement both increase β1 integrin-mediated adhesion (18, 19), ligation of these co-stimulatory markers has markedly different effects on T cell migration. Engagement of CD28 enhances the migrational capacity of T cells into inflamed tissue whereas ligation of CTLA4 inhibits T cell recruitment (20). However, the underlying mechanisms of these opposing effects are unknown. CD28 controls migration through upregulation of OX40, which is instrumental for CXCR5 expression and T cell localization to germinal centers (21). Co-stimulation by CD28 in combination with strong TCR signaling activates the PI3K/AKT pathway, a key regulator of glucose metabolism, which together with the mammalian target of rapamycin (mTOR) orchestrates the energy demands necessary for effector development (22). The PI3K/AKT and mTOR pathways not only regulate the necessary metabolic changes to the T cell, but also regulate their migratory capacity. Specifically, mTOR and Akt activation inhibits the Foxo family of transcription factors leading to decreased expression of kruppel-like factor 2 (KLF2), which in turn leads to the reduced expression of CD62L, the IL-7 receptor, and CCR7 (23–26). Importantly for the ability of cells to leave the LN, KLF2 also regulates the expression of S1P1 promoting the egress of T cells into the circulation (27).

CCR7 and CD62L expression may also be impacted by signaling via the co-stimulatory molecule ICOS, which is a member of the CD28-superfamily and expressed on activated T cells. Ligation of ICOS was demonstrated to down-regulate CCR7 and CD62L after activation, leading to more efficient migration of CD4+ T cells into the lungs and a reduced return to the LN (28).

Whether other co-stimulatory pathways link migratory capacity and T cell activation has not yet been defined, but activated T cells express high levels of multiple MR, and engagement of MR themselves can provide co-stimulation. Early participation of T cells in a response, when antigen levels and co-stimulatory signals are high, leads to loss of CD62L and increased expression of CD44, PSGL-1, S1P1, and the integrins LFA-1 and/or α_4_β_1_ (VLA-4) as well as other integrins that engage the extracellular matrix (ECM) (29). LFA-1 contributes to T cell responses by enhancing TCR signaling, production of IL-2, and proliferation, but also modulates T cell polarization and motility (11). α_4_β_1_ can also contribute to Th1 development by acting as a co-stimulatory molecule (30). In addition, pro-inflammatory cytokines can rapidly upregulate CD44 and its ability to bind its ligand, the ECM component hyaluronic acid (HA) (31). DC can synthesize and bind HA and during naïve T cell-DC interactions, ligation of CD44 by HA can enhance T cell cytokine production and proliferation by T cells that have received signals through the TCR (32). Similarly, ligand binding capacity is induced on PSGL-1 in response to T cell activation. In our own work, we find that CD4+ Th1 effector cells are heterogeneous with respect to expression of functional PSGL-1. Specifically, those cells with the highest levels of functional PSGL-1 are the most proliferative effectors with the greatest capacity for effector cytokine secretion and for cytotoxic activity (Bradley and Swain, unpublished observations).

## DC-Derived Cytokines Drive Distinct MR Expression Profiles in T Cell Subsets

DC further influence T cell heterogeneity by virtue of the cytokines they secrete (3). As the immune response progresses, exposure of activated CD4+ T cells to polarizing cytokines leads to the development of subsets variously defined by function and transcription factor expression. These include the well-defined Th1, Th2, Th17, Tfh, and Treg subsets and the less understood Th9 and Th22 subsets, as well as CD4+ cells with cytotoxic activity, ThCTL (33). Cytokines released by DC work in part by inducing particular transcriptional profiles in T cells that promote expression of effector cytokines, but they also induce the expression of MR that allow for microanatomical localization (Figure 1, panel 2). For example, T cell activation is associated with the expression of the transcription factors Blimp-1 as well as T-bet. T-bet expression is sustained by DC-derived IL-12 and determines a CD4+ Th1 and CD8+ transcriptional program, which allows effector T cells to produce IFN-γ (33). T-bet induction is accompanied by the expression of CXCR3 (34) that binds the chemokines CXCL9, CXCL10, and CXCL11, which are frequently associated with inflammation. Asymmetric division can be associated with differential partitioning of T-bet (35, 36) and might also contribute to effector cell heterogeneity. This concept has been predominantly studied in terms of the generation of effector T cells that give rise to subsets memory cells that differentially express CD62L/CCR7 (see Role of Location and MR Signaling on the Effector to Memory T Cell Transition). Tfh cells develop concurrently with Th1 and CD8+ effector cells, regulated by induction of the transcription factor Bcl6 (with loss of Blimp-1) (37), which results in the downregulation of PSGL-1 (38), loss of CCR7, and increased expression of CTLA4 and CXCR5. The expression of CXCR5 results in increased responsiveness to CXCL13 and allows Tfh cells to migrate into B cell follicles in SLO (39).

Thus, changes in MR expression define unique phenotypes that can play critical roles in the migration and retention of effector cells. The combined outcome of TCR engagement of MHC in the context of peptide, signaling via co-stimulatory molecules, and access to polarizing cytokines determines in part the heterogeneity in MR expression profiles on T cell, which has a significant effect on their ability to exert their effector function.

## Distinct MR Expression Profiles Determine Tissue-Specific Migration and Effector Function

**Figure 2:**
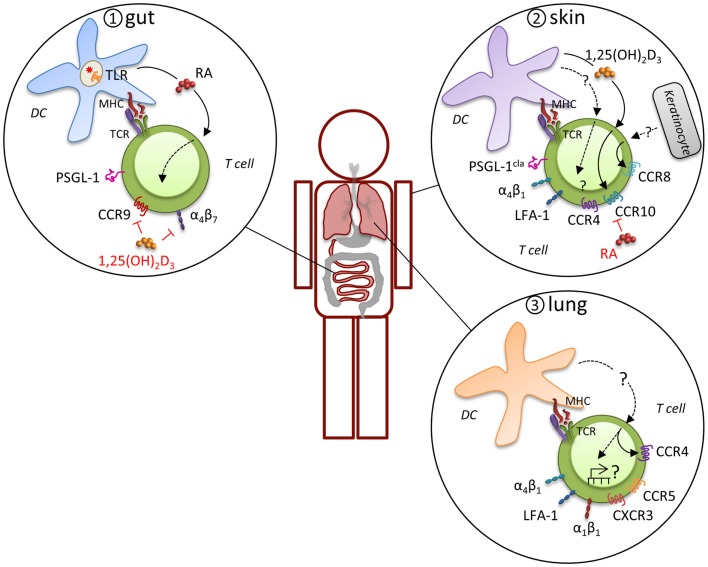
**Induction of tissue-specific MR profile**. Expression of distinct MR combinations results in tissue-specific migration. T cells that are primed in gut-associated lymphoid tissues preferentially express functional PSGL-1, α_4_β_7_, and CCR9, which support migration through the post capillary venules of the small intestine to enter the lamina propria and intraepithelial compartment (panel 1). CD103-expressing DC impart the intestinal homing signature on T cells during priming by expression of retinoic acid (RA) in response to microbiota-driven toll-like receptor (TLR) signaling. A different program of MR usage is induced during priming of naïve T cells in skin-draining LN (panel 2). T cells in the skin express the cutaneous lymphocyte antigen (CLA), an inducible carbohydrate modification of PSGL-1, CCR4, CCR8, CCR10, α_4_β_1_, and LFA-1, which mitigates their migration into the skin. Analogous to intestinal imprinting, CCR10, but not CCR4, expression is regulated by skin-draining DCs that synthesize the vitamin D3 metabolite, 1,25(OH)_2_D_3_. 1,25(OH)_2_D_3_ suppresses α_4_β_7_ and CCR9 expression and RA inhibits CCR4 and CCR10 expression. In addition, CCR8 expression is imprinted by epidermal keratinocytes, although the skin-specific factors that induce CCR8 remain unknown. Evidence for imprinting of T cells in the lung is limited, but recent evidence suggests that lung DC-activated T cell migrate more efficiently into the lung, which was attributed to CCR4, although other MR are likely to contribute (panel 3). Many lymphocytes in the lung express high levels of α_4_β_1_, α_1_β_1_, and LFA-1 and CD8+ T cells primed in the mediastinal LN are enriched for CCR5 and CXCR3 expression, suggesting a pulmonary profile driven by distinct molecular mechanisms.

In general, effector T cells express a variety of MR that may be used alternatively or in combinations for migration and retention at sites of inflammation, which has led to the concept that expression of distinct MR combinations results in tissue-specific migration (Figure 2). For example, there is considerable evidence that a specialized program develops in T cells that are primed in gut-associated lymphoid tissues (mesenteric LN and Peyer’s patches) that directs migration into the gut mucosa. Effector T cells in these sites preferentially express functional PSGL-1, α_4_β_7_, and CCR9, which support migration via P-selectin, MAdCAM-1, and CCL25, respectively, through the post capillary venules of the small intestine to enter the lamina propria and intraepithelial compartment (40, 41). CD103 (integrin α_E_)-expressing DC impart the intestinal homing signature on T cells during priming, although CD103-positive and -negative DC populations can both prime T cells (42, 43). The induction of α_4_β_7_ and CCR9 is driven by retinoic acid, which is specifically synthesized by gut-associated CD103-positive DC, but not by CD103-negative or extra-intestinal DC (44). This capacity of intestinal CD103-positive DC to produce retinoic acid is acquired via toll-like receptor signaling, presumably in response to interactions with the microbiota (45). In addition to CD103-positive DC, the stroma of the mesenteric LN also expresses high levels of retinoic acid-producing enzymes and may have a considerable effect on the intestinal homing signature of T cells (46). T cell migration to the gut epithelium leads to TGF-β-dependent induction of α_E_β_7_ on the T cells, which contributes to the retention of both CD4+ and CD8+ effectors by engagement of epithelial cell-expressed E-cadherin. Once an effector response subsides, residual CD8+ T cells can remain in the epithelial compartment as memory cells by this mechanism. CD4+ T cells are primarily maintained in the lamina propria, where they may require the continued local exposure to antigen for persistence.

A different program of MR usage is induced during priming of naïve T cells in skin-draining LN under inflammatory conditions. Both migrating Langerhans cells and conventional DC can present antigens to naïve T cells (47). Upon activation, these T cells express the cutaneous lymphocyte antigen, an inducible carbohydrate modification of PSGL-1 that preferentially binds to E-selectin on the endothelium of inflamed skin (48, 49). CD43 and CD44 expressed by T cells can also act to engage E-selectin. Although many chemokines can participate in migration of effector T cells to the skin depending on the characteristics of the infection or inflammation (50), the capacity for recruitment of effector T cells to the skin is primarily associated with expression of CCR8 and CCR10. While skin T cells express high levels of CCR4 and its ligands CCL17 and CCL22 are produced by epidermal keratinocytes and dermal fibroblasts, skin DC have not been shown to imprint CCR4 expression, which suggests that this MR is not exclusively a skin-homing molecule. Conversely, CCR8 expression on T cells is imprinted by epidermal keratinocytes, although the skin-specific factors that induce CCR8 remain unknown (51). CCR10 is also acquired by effector CD8+ T cells that migrate to skin in response to the epidermal chemokine CCL27. Analogous to intestinal imprinting, CCR10 expression is regulated by skin-draining DCs that synthesize the vitamin D3 metabolite, 1,25(OH)_2_D_3_ (52). While1,25(OH)_2_D_3_ suppresses α_4_β_7_ and CCR9 expression, it is not sufficient to induce a *bona fide* skin-homing program (i.e., other skin-homing receptors are not induced). Interestingly, retinoic acid inhibits the generation of T cells expressing these skin-homing receptors. The integrins LFA-1 and α_4_β_1_ can both mediate transmigration from the blood into the skin through engagement of their respective ligands, ICAM-1 and VCAM-1 (53). Expression of the α_E_β_7_ integrin is also highly associated with skin-homing effector T cells and is thought to account for retention of effector cells that can make a transition to memory *in situ* [(54), see below].

While specific MR phenotypes that allow directed migration to the intestinal mucosa or skin have been identified, evidence for distinct MR expression patterns for the respiratory system has been lacking. In a recent paper, CD4+ and CD8+ T cells that were activated specifically by lung DC were shown to home more efficiently into the lung in response to inhaled antigen (55). CCR4 contributed to the lung-homing advantage of lung DC-activated CD4+ T cells and to reduced morbidity *in vivo* in response to influenza virus infection. However, this was a partial effect and it is likely that other MR are induced by lung DC. For example, we demonstrated that numerous genes involved in cell migration and motility are differentially expressed between lymphocytes responding in the SLO versus the lung (56) and many lymphocytes in the lung express high levels of α_4_β_1_, α_1_β_1_ (CD49a, VLA-1), and LFA-1 (57–61). In addition, CD8+ T cells primed in the lung-draining mediastinal LN by DC are enriched for CCR5 and CXCR3 expression compared to T cells primed in other sites (62) and these receptors regulate T cell effector responses and their contraction after infections with influenza viruses or *Mycobacterium tuberculosis* (Mtb) (63). Thus, it is tempting to speculate that some MR contribute to a specific pulmonary profile driven by distinct molecular mechanisms (see also Role of Location and MR Signaling on the Effector to Memory T Cell Transition).

A critical factor that can also impact T cell migration to particular sites is the changes to the site mediated by the pathogen and the inflammation initiated by the pathogen. Inflammation can alter the functional activity of the stromal cells thereby impacting recruitment from the circulation. After influenza virus infection, the ThCTL subset and ThCTL-associated genes are observed almost exclusively in the lung (56, 64, 65) suggesting a role for virus-specific inflammation in coordinating migration and function. For effective immunity to influenza virus, activated CD8+ T cells need to interact with pulmonary DC in an antigen-specific manner at the site of inflammation and while the impact on MR phenotype was not studied, this interaction could introduce additional heterogeneity (66). Following infection with Mtb, the infected site becomes dominated by macrophages before the arrival of T cells, thereby creating an environment very different from the normal lung (67). Indeed as disease, develops T cell migration into the lung and within the inflamed site appears to be regulated by the development of B cell follicles and the expression of CXCL13 (68, 69). We have demonstrated that within the chronically inflamed Mtb-infected tissue, activated effector T cells express high levels of α_4_β_1_ and that this is regulated by the presence of nitric oxide (70). This brings to prominence the idea that the inflammatory site, particularly the presence of inflammatory macrophages, can influence the expression of MR and thereby influence function and persistence of T cells.

It is important to bear in mind that tissue-specific responses can be re-programed by the exposure of T cells to alternative environments. For example, in a tumor model in which the site of implantation led to CD8+ T cell priming in different LN, different patterns of expression of integrins and selectin ligands were acquired by dividing cells in different sites that were associated with differential homing (71). Another study showed that CD4+ T cells primed in the gut-associated mesenteric LN by oral immunization acquired a skin-homing phenotype when transferred to recipients immunized subcutaneously (72). In addition to the significant role of DC imprinting on the acquisition of tissue-specific homing, DC trafficking itself has a substantial impact on T cell migratory heterogeneity [reviewed in Ref. (73)]. In conclusion, the function and location of effector T cells is governed by the expression of distinct MR combinations that are regulated by the environment. The combination of cell types and chemokines that are induced in response to inflammation can dramatically alter the environment within the tissue, which can significantly alter the MR phenotype and influence T cell migration. This suggests that phenotypes with respect to MR expression can be dynamically modulated for therapeutic purposes at particular sites to maximize vaccine efficacy and protection from invading pathogens.

## MR Signaling Directs Movement of T Cells into and within Peripheral Tissues

MR-mediated localization of cells from blood into tissue is well-studied. Once activated in SLO, T cells are released into the circulation, a process that is regulated by the presence of high concentrations of S1P, in the lymphatic vessels (29). Once in the circulation, effector T cells are directed to sites of inflammation by activated endothelium. Pro-inflammatory mediators including TNF-α, IL-1, and IL-6 can rapidly activate endothelial cells to upregulate expression of selectins and CD44 (5). The latter binds to its ligand HA that is liberated as fragments from the ECM in response to inflammation [reviewed in Ref. (74)]. These molecules can be functionally redundant with respect to migration and individually regulate T cell encounters with the luminal surface of the vasculature through engagement of PSGL-1 or E-Selectin ligand. Chemokines, presented or secreted by endothelial cells as a consequence of the innate response, induce signaling via G protein-coupled receptors that upregulate the adhesiveness of integrins for their counter receptors that include ICAM-1, VCAM-1, and MadCAM-1 [reviewed in Ref. (5)].

Much less is known regarding the mechanisms that are engaged to mediate movement of cells within tissues. Upon extravasation, T cells must traverse the basement membrane, which is achieved by activation of matrix metalloproteases (MMPs). MMPs facilitate migration into and within the tissue interstitium by proteolytic degradation of ECM components. T cells predominantly produce the gelatinases MMP2 and MMP9 that cleave type IV collagen (75). We and others find that different T cell subsets express different amounts of MMP2 or MMP9 *in vitro* [Baaten, unpublished observation, (76, 77)]. It is unclear what induces activity *in vivo* and what causes their differential induction in these subsets, but the numerous cell–cell contact-dependent interactions via selectins, CCRs, and integrins during the extravasation process could impact MMP expression and/or activity. Initial interactions with the endothelial layer could upregulate MMP expression in T cells during transmigration (78–81). MMP2 and MMP9 production in T cells can be induced by ligation of integrins (α_L_β_2_, α_4_β_1_, α_5_β_1_, α_V_β_1_) following interaction with the endothelial layer (e.g., ICAM, VCAM) or constituents of the basement membrane (e.g., fibronectin) (80–86). Depending on the type of integrin, differential MMP expression can be induced in T cells (80). Thus, MR heterogeneity could impact T cell motility by regulating MMP activity. In addition, integrins are able to bind MMP2 and MMP9, which has been proposed to target catalytic activity to specific substrates within the pericellular space and assist cellular invasion (87, 88). However, the *in vivo* function of gelatinase activity for basement membrane degradation remains controversial and proteolytic cleavage of both ECM and non-ECM substrates can regulate migration and motility through other mechanisms (89). For example, chemotactic factors or MR expression could be altered by MMP-mediated proteolysis (90) thereby modulating T cell migration. More studies are required to identify how the molecular events that regulate rolling and tethering affect the phenotype and function of migrating T cells during transmigration.

To achieve directed migration, T cells undergo cytoskeletal rearrangements that allow for the formation of a polarized shape with a leading edge and a uropod that remains in contact with the ECM (91, 92). This process requires multiple signaling events that translate signals from membrane proteins into cell movement. Actin polymerization, regulated by members of the Rho GTPase family (93), controls forward movement of the cell at the leading edge where CCRs (94), integrins, and the TCR are located. The cell organelles, microtubule organizing center, and Golgi apparatus, as well as most MR, including PSGL-1, CD44, CD62L, and ICAM-1, become localized in the uropod. These receptors contribute to the signaling processes that regulate cell migration by binding ezrin and moesin, which anchor MR to the actin cytoskeleton and support the formation of clusters of MR. ERM proteins are activated through PI3K signaling to associate with the cytoplasmic domains of MR in the uropod, where they can serve as adaptor proteins for signal transduction. There can be considerable crosstalk between MR in response to signaling. For example, ligation of either PSGL-1 or CD44 can be associated with PI3K activation. Functional PSGL-1 also requires recruitment of ERM proteins to transmit signals necessary for adhesion (95). The activation status of ERM proteins is positively regulated by RhoA (93) that signals through the Rho-associated kinase, ROCK, which has critical roles in cell division and cell survival (96). Rho activation is associated contraction of the uropod that enables the forward movement of the cells (97).

The signaling pathways engaged by MR to mediate cell movement can profoundly modulate effector T cell responses and fitness for proliferation and survival. During T cell movement within tissues, signaling pathways continue to be dynamically regulated by engagement of the ECM via CD44 and integrins. For example, in a tumor model, *Cd44*-deficient CD8+ effector T cells could not maintain polarity and as a consequence had impaired cytotoxic activity (98). Recently, the integrin α_V_ was demonstrated to be involved in context-dependent motility of Th1 cells in inflamed skin, and its expression was crucial for pathogen clearance (99). During migration within tissues T cells interact with other migrating and non-migrating cells including DC and tissue macrophages, which can present antigens and produce cytokines to enhance the effector T cell response. For instance, after influenza virus infection, DC elicit both cytotoxic activity and cytokine production that was dependent upon co-stimulation via CD80 and CD86, whereas engagement of epithelial cells that lack these receptors selectively stimulated cytotoxicity (100), revealing that effector cells can display heterogeneity depending on contextual cues.

## Role of Location and MR Signaling on the Effector to Memory T Cell Transition

The hallmark of immunological memory is a quicker and more effective response upon re-encountering a pathogen. Migratory capacity can be intimately associated with effector function of responding cells at sites of inflammation that have the potential to become memory cells. MR contribute significantly to the functionality of memory T cells by enabling migration for surveillance, motility for *in situ* positioning, and signal interpretation from the ECM that enable cell survival. The timing of migration of naïve T cells into an inflamed LN can impact the formation of memory. Naïve T cells that enter LN later during the immune response are more likely to become central memory T cells (101, 102). These T cells receive less stimulation and consequently are less likely to adopt the migratory phenotype characterized by loss of CD62L and CCR7, and upregulation of integrins and PSGL-1. Strength of signaling also impacts the PI3K/Akt-mTOR pathway (see above) that can also influence the development of memory. Inhibition of this pathway limits differentiation of effector CD8+ T cells and results in greater generation of central memory CD8+ T cells during acute lymphocytic choriomeningitis virus (LCMV) infection (23). Memory T cells that preserve expression of CD62L and CCR7 maintain the migration patterns of naïve cells through SLO, allowing them to maximize the chance of finding antigen-specific DC early during a re-infection. These T cells also maintain expression of IL-2, a key cytokine for optimal memory cell responses [Swain, unpublished observations, (103, 104)].

Although the majority of effector T cells responding to pathogens in non-lymphoid tissues lack naïve T cell MR expression, CCR7 distinguishes T cells with the capacity to egress from non-lymphoid organs, such as the skin and lung (105, 106). While most effector cells in the lung die by apoptosis, some responding T cells can undergo regulated egress via the lymphatics and return to the circulation by engagement of the CCR7 ligand CCL21, which is expressed on lymphatic endothelium. Since effector CD4+ T cells responding to lung inflammation can be partitioned into subsets by expression of CCR7 (107), it is possible that these represent functionally distinct effectors with those capable of egress having a greater potential for survival and effector memory formation in SLO. Interestingly, the absence of CCR7 is associated with effector cell accumulation in the lung in allergic inflammation, suggesting that CCR7-regulated egress from the lungs may be an important factor in terminating the effector response (108). In addition, S1P may contribute to the regulation of egress from non-lymphoid tissues (109). However, some effector T cells express CD103 and are programed to persist in non-lymphoid sites. These effector memory T cells are known as tissue resident memory cells that are thought to provide site-specific protection against repeat infections (110). Their localization and persistence is mediated by MR to facilitate the accelerated response to re-encounter with an antigen.

Although MR expression predicts differences in migration of memory T cells to SLO and peripheral tissues, it is now becoming clear that this can have important functional consequences. In the skin, the anatomical localization and migration pattern of CD4+ and CD8+ memory T cells are substantially different following epicutaneous infection with herpes simplex virus as a result of distinct migratory programing in these two subpopulations (111). Whereas CD103+ CD8+ memory T cells remained in the epidermis as tissue resident memory cells, CD4+ effector memory T cells resided in the dermis, but were highly mobile and able to recirculate due to expression of E/P-selectin ligands. Whether this migration-linked T cell subset specialization is further impacted by T cell heterogeneity within the subsets is unknown. Nevertheless, signals received from the ECM via MR *in situ* can modulate the effector program to increase the potential for surviving the contraction phase of an immune response to enable transition to memory. For example, CD8+ and CD4+ T cells infiltrating the lung can differentially express α_1_β_1_ and α_2_β_1_ (CD49b, VLA-2), respectively (112). As a result, the different subsets localize to different areas within the lungs: CD8+ T cells that preferentially express α_1_β_1_ locate near the basement membranes of either the airways or blood vessels, whereas α_2_β_1_ CD4+ T cells primarily localize within the interstitial spaces. After influenza virus infection, α_1_β_1_-binding of collagen allows memory T cells to persist and function in the lungs (59, 113, 114). Similarly, signaling via CD44 is necessary for the survival of Th1 effector cells in lungs and the transition to memory (115). We and others find that acquisition of HA binding capacity by CD8+ T cells distinguishes effector cells with a greater potential to form memory cells [Bradley, unpublished observations, (116)].

It is becoming increasingly appreciated that microanatomical location can be critical in determining the signals that T cells receive during activation (86), but perhaps also during homeostasis and transition to memory. For example, T cells that are in proximity to the lymphatic vessels in the LN have ready access to antigens and pro-inflammatory cytokines that drain from sites of infection (117). In some viral and bacterial infections, notably systemic infections with LCMV (118) or *Listeria monocytogenes* (119), potent, but short-lived CD8+ effector T cells are distinguished by high expression of KLRG1 and low levels of IL-7Rα, whereas effectors with the reciprocal phenotype are better able to form memory cells. During acute infection with LCMV, short-lived effector CD8+ T cells are localized in the red pulp, whereas memory precursors preferentially locate to the white pulp in contact with stromal cells that produce IL-7 (120). During chronic LCMV infection, which leads to T cell exhaustion with sustained expression of the inhibitory receptor, programed death-1 (PD-1), both CD4+ and CD8+ effector T cells locate in the red pulp where their motility is impaired by PD-1 engagement of PD-L1 on macrophages and DC (121). However, in acute infections PD-1 plays a role in preventing terminal Th1 and CD8+ effector cell differentiation following pathogen clearance (122), highlighting that the integration of positive and negative signals is crucial for both the development of functional effector cells and memory. These examples underscore that a coordinated interplay of signaling via MR and the cytoskeleton is integral to T cell responses and survival within tissues and demonstrate the complexity and differences of CD4+ and CD8+ memory T cell responses and suggest that interactions with the ECM at the site of infection could have a significant impact on T cell function. By extension it is likely that MR signals play roles in the micro-location of effector and memory subsets that enable them to receive optimum homeostatic signals.

## Conclusion

The ability of T cells to move around the body is crucial for their effector function and immunity to infection with pathogens. CD4+ and CD8+ T cells are heterogeneous and subsets have been defined based on the expression of CCRs, selectins, and integrins that are able to engage inflamed endothelium, the ECM, and cells of the innate immune system. For effector T cells that relocate to sites of inflammation, considerable plasticity in their responses can occur during the priming process with DC that imparts a tissue-specific MR phenotype to ensure efficient homing of activated T cells to the correct tissue. There are many mechanisms that contribute to control of T cell migration, motility, and egress within tissues and the coordinated interplay of these signals are only now becoming appreciated as crucial regulators of T cell function. MR are able to provide important contextual information from the ECM that allow for distinct transcriptional profiles that dictates not only cell polarity and interstitial motility, but also effector function, cell survival, and transition to, and maintenance of memory. Thus, MR heterogeneity has a direct impact on T cell immunity and protection from invading pathogens. The identification of additional distinct tissue profiles and the molecular mechanisms that control them have direct therapeutic relevance.

## Conflict of Interest Statement

The authors declare that the research was conducted in the absence of any commercial or financial relationships that could be construed as a potential conflict of interest.
